# Identification and validation of autophagy-related genes in Kawasaki disease

**DOI:** 10.1186/s41065-023-00278-9

**Published:** 2023-04-21

**Authors:** Hao Zhu, Biao Xu, Cunshu Hu, Aimin Li, Qing Liao

**Affiliations:** 1 Department of Pediatrics, Jingzhou Hospital Affiliated to Yangtze University, Jingzhou, China; 2 Department of Pediatrics, Xianning Central Hospital, The First Affiliated Hospital of Hubei University of Science and Technology, Xianning, China; 3 XianNing Public Inspection and Testing Cente, Xianning, China

**Keywords:** GEO database, Kawasaki disease, Autophagy, Bioinformatics, qRT-PCR

## Abstract

**Background:**

Kawasaki disease (KD) is a systemic vasculitis of unknown etiology affecting mainly children. Studies have shown that the pathogenesis of KD may be related to autophagy. Using bioinformatics analysis, we assessed the significance of autophagy-related genes (ARGs) in KD.

**Methods:**

Common ARGs were identified from the GeneCards Database, the Molecular Signatures Database (MSigDB), and the Gene Expression Omnibus (GEO) database. ARGs were analyzed by Gene Ontology (GO) and Kyoto Encyclopedia of Genes and Genomes (KEGG) enrichment analysis and protein–protein interaction (PPI) network analysis. Furthermore, related microRNAs (miRNAs), transcription factors (TFs), and drug interaction network were predicted. The immune cell infiltration of ARGs in tissues was explored. Finally, we used receiver operating characteristic (ROC) curves and quantitative real-time PCR (qRT-PCR) to validate the diagnostic value and expression levels of ARGs in KD.

**Results:**

There were 20 ARGs in total. GO analysis showed that ARGs were mainly rich in autophagy, macro-autophagy, and GTPase activity. KEGG analysis showed that ARGs were mainly rich in autophagy—animal and the collecting duct acid secretion pathway. The expression of WIPI1, WDFY3, ATP6V0E2, RALB, ATP6V1C1, GBA, C9orf72, LRRK2, GNAI3, and PIK3CB is the focus of PPI network. A total of 72 related miRNAs and 130 related TFs were predicted by miRNA and TF targeting network analyses. Ten pairs of gene–drug interaction networks were also predicted; immune infiltration analysis showed that SH3GLB1, ATP6V0E2, PLEKHF1, RALB, KLHL3, and TSPO were closely related to CD8 + T cells and neutrophils. The ROC curve showed that ARGs had good diagnostic value in KD. qRT-PCR showed that WIPI1 and GBA were significantly upregulated.

**Conclusion:**

Twenty potential ARGs were identified by bioinformatics analysis, and WIPI1 and GBA may be used as potential drug targets and biomarkers.

**Supplementary Information:**

The online version contains supplementary material available at 10.1186/s41065-023-00278-9.

## Introduction

Kawasaki disease (KD) is a vasculitis that primarily affects the coronary arteries [1]. It can cause acquired heart disease in children [2]. Previous studies suggested that 25% of KD patients are not timely diagnosed and treated, leading to coronary artery disease (CAD) and possibly even sudden death [3]. However, the pathogenesis of KD is not clear. It is currently considered to be related to inflammatory, immune, and genetic factors. Further study of its pathogenesis is necessary to facilitate early diagnosis and develop better treatment strategies.

Autophagy is an evolutionarily conserved catabolic process that maintains the dynamic balance of the internal environment [4]. Under the regulation of autophagy-related genes (ARGs), intracellular bilateral membrane structures encapsulate substances and organelles to be degraded and thereby form autophagosomes, which then fuse with lysosomes, forming autolysosomes, which perform cellular metabolism through the action of hydrolytic enzymes [5]. A previous study has shown that autophagy is related to inflammatory diseases, cardiovascular diseases, neurodegenerative diseases, and cancer [6]. KD can easily damage vascular endothelial cells and cardiomyocytes, and one study has reported that KD may lead to vascular endothelial cell injury through autophagy [6]. Mouse experiments have confirmed that inhibition of autophagy can protect the vascular endothelium [7]. Although high-throughput sequencing is often employed to identify disease targets, the relationship between KD and autophagy still needs to be further explored.

In the present study, we identified ARGs and conducted Gene Ontology (GO) and Kyoto Encyclopedia of Genes and Genomes (KEGG) enrichment analysis. In addition, we constructed a protein–protein interaction (PPI) network of differentially expressed genes (DEGs) between the KD and control groups using the STRING database, we predicted ARG-targeted microRNAs (miRNAs) and transcription factors (TFs), and we constructed a gene–drug interaction network. Moreover, we examined immune cell infiltration in KD, and the expression levels of ARGs were analyzed in KD patients and healthy controls. In conclusion, we analyzed the roles of ARGs in KD to understand the pathogenesis of KD.

## Materials and methods

### Data acquisition and processing

We acquired the KD-related datasets GSE68004 [8] and GSE73461 [9] from the Gene Expression Omnibus (GEO) database (https://www.ncbi.nlm.nih.gov/geo/) [10] via the GEOquery package [11]. The Homo sapiens dataset GSE68004 was obtained from the data platform GPL10558, containing a total of 162 samples, comprising 37 control and 125 KD samples. We eliminated samples that were positive for adenovirus, group A Streptococcus, and/or other diseases from the dataset. For the subsequent analysis, 55 KD samples and 37 controls were used. The H. sapiens dataset GSE73461 was obtained from the data platform GPL10558, containing a total of 459 samples, comprising 55 control and 404 KD samples. We removed samples that were positive for bacterial infections, viral infections, inflammatory diseases, and/or uncertain bacterial or viral etiological infections, as well as samples containing missing data. For the subsequent analysis, 77 KD samples and 55 control samples were used. We corrected and normalized the datasets GSE68004 and GSE73461 by the R package limma [12].

### Identification of ARGs

Based on the annotation information of GSE68004 and GSE73461 samples, we divided the samples into normal and KD groups. We employed the R package limma to perform differential expression analysis of genes in different groups, setting |log(fold change)|> 1 and Padj < 0.05 as the threshold criteria. Genes with log(fold change) > 1 were upregulated, and genes with log(fold change) <  − 1 were downregulated. The differential expression analysis results were visualized using the R package pheatmap, and heatmaps and volcano maps were plotted using the R package ggplot2. We took the intersection of genes that were differentially expressed in both datasets for subsequent analysis.

The GeneCards database (https://www.genecards.org/) [13] provides comprehensive information about human genes. The Molecular Signatures Database (MSigDB) [14] contains over 6700 datasets from the canonical pathway and publication experimental characterization collections. We downloaded the list of autophagy-associated genes from the GeneCards database and the annotated gene set of MSigDB using the term “autophagy” as a search term. We obtained 7236 genes associated with autophagy from the GeneCards database and 505 genes associated with autophagy from the MSigDB database. We intersected the sets of ARGs from different databases and obtained a total of 473 genes. We obtained the DEGs associated with autophagy in KD by intersecting the genes that were differentially expressed in both datasets with the genes associated with autophagy.

### GO and KEGG enrichment analysis

GO annotation analysis [15] is a common method for enrichment studies. The KEGG database [16] contains information about genomes and biological pathways. GO annotation analysis and KEGG pathway enrichment analysis of DEGs were conducted using the R package clusterProfiler [17]. *P* < 0.05 was considered to indicate statistical significance.

### Construction of a PPI network and hub gene identification

The STRING database (https://cn.string-db.org/) [18] can be used to predict PPI networks. A PPI network was built between the KD and normal groups using the STRING database with a coefficient of 0.4. We exported PPI results, visualized them using Cytoscape software [19], and screened the genes in the top 10 PPI relationships using the cytoHubba plugin [20].

### Construction of mRNA–miRNA and mRNA–TF networks

To comprehensively and accurately mine the regulatory relationships between mRNAs and miRNAs and between mRNAs and TFs in KD, we predicted the target miRNAs of ARGs in KD according to the miRWalk [21], miRDB [22], miRTarBase [23], and starBase [24] databases. miRNAs identified in all four databases were considered to be targeted miRNAs of ARGs in KD. The target TFs of ARGs in KD were predicted based on the hTFtarget [25] and knockTF databases, and TFs identified in both databases were considered to be targeted TFs of ARGs in KD. We constructed miRNA–mRNA and mRNA–TF interaction networks via Cytoscape.

### Construction of target gene–drug networks

The Drug–Gene Interaction Database (DGIdb) version 3.0 [26] is an open-source project that helps users mine existing resources and generate hypotheses about how genes can be used as targets for drug development. To explore the interactions between genes and drugs, we constructed a gene–drug network for autophagy-related DEGs in KD using the following parameter settings: Preset Filters, FDA Approved; Advanced Filters, Source Databases, 22 of 22; Gene Categories, 41 of 41; Interaction Types, 51 of 51. Cytoscape software was used to visualize the gene–drug network.

### Immune cell infiltration analysis

CIBERSORT [27] was used to analyze the proportions of different immune cell types based on gene expression data. We used the R software CIBERSORT package for the gene expression matrix data, combined with the LM22 signature gene matrix, and filtered the output samples with *P* < 0.05 to derive the immune cell infiltration matrix. Heatmaps were generated using the R package ggplot2 to show the infiltration of the 22 immune cell types in each sample.

### Quantitative real-time PCR (qRT-PCR)

From March 2022 to September 2022, we collected 10 whole blood samples, comprising five KD and five healthy control samples, from the Xianning Central Hospital of Hubei, China. Samples were from patients aged 8 months to 6 years. We collected 3–5 mL whole blood samples in the tubes containing EDTA from all the participants and submitted them to white blood cell (WBC) enrichment. Total RNA was extracted from leukocytes of children with KD and healthy controls, and blood samples from KD children were collected before intravenous immunoglobulin (IVIG) and aspirin treatment. Reverse transcription was performed using the Servicebio® RT First Strand cDNA Synthesis Kit (Servicebio, Wuhan, China). Quantitative PCR (qPCR) was performed using 2 × SYBR Green qPCR Master Mix (None ROX) (Servicebio, Wuhan, China) following the manufacturer’s instructions. The thermocycling conditions were as follows: initial activation at 95 °C for 30 s, followed by 40 cycles at 95 °C for 15 s, 60 °C for 30 s, and 60 °C for 30 s. GAPDH was used as the internal reference for data normalization. Relative expression was calculated by the 2 − ΔΔCt method. Primers are listed in Table S1.

### Statistical analysis

Receiver operating characteristic (ROC) curve analysis was conducted to predict dichotomous outcome events with continuous variables and to evaluate the degree of good or bad predictor variables. We plotted ROC curves using the R package pROC [28] and calculated the area under curve (AUC) values to assess the diagnostic performance of each KD ARG. AUC > 0.9 indicates that the gene has a good diagnostic effect. All calculations and statistical analyses were performed using R (https://www.r-cret.org/, version 4.0.2). For the comparison of continuous variables between the two groups, the statistical significance of normally distributed variables was estimated by the independent Student t-test, and the differences between non-normally distributed variables were analyzed by the Mann–Whitney U test (i.e., Wilcoxon rank sum test). All statistical tests were two-sided. Differences were considered statistically significant at *P* < 0.05.

## Results

### Identification of genes associated with autophagy in KD

**Fig. 1:**
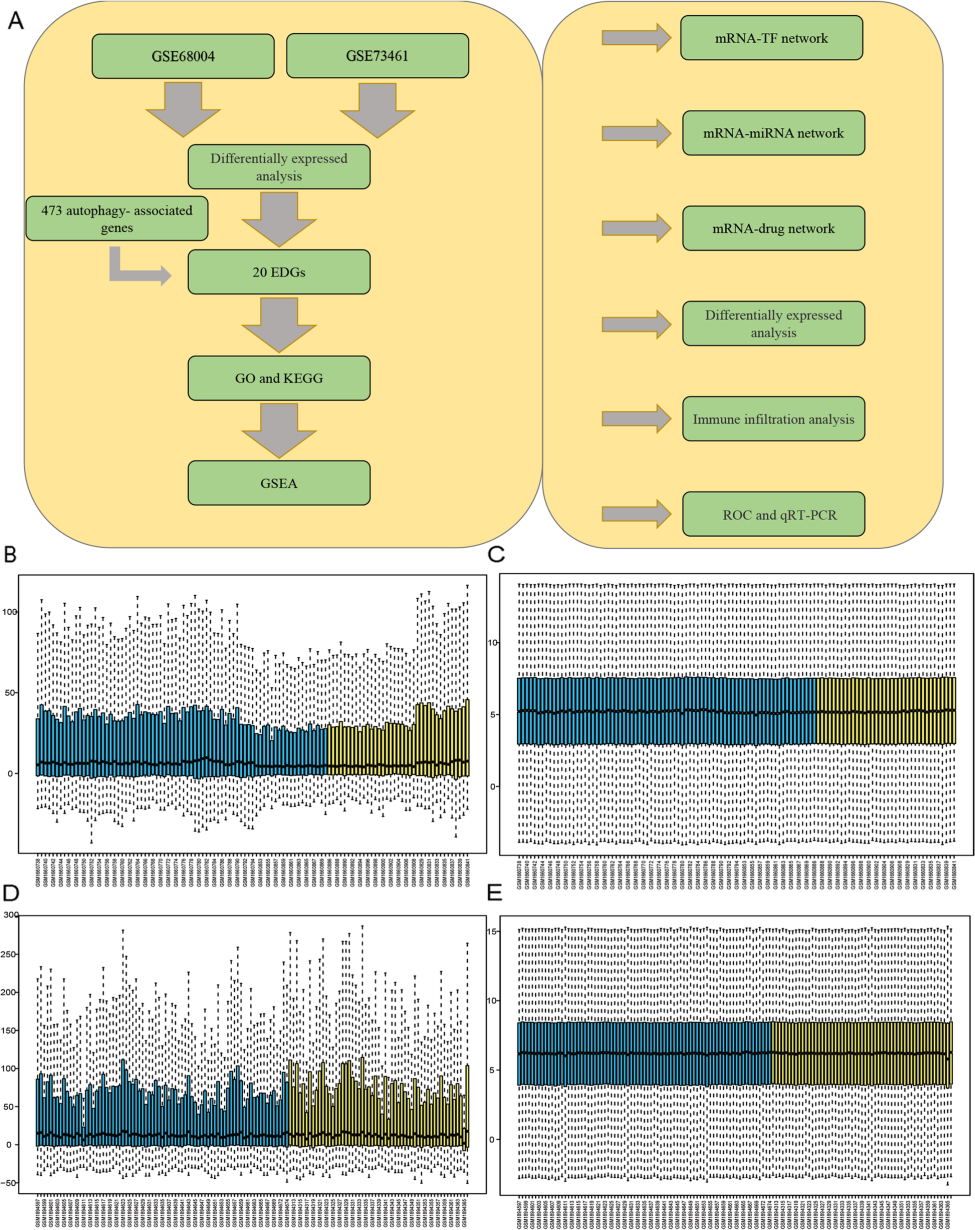
Study flowchart and box diagram of data correction. **A** Study flowchart. **B**, **C** Boxplot of dataset GSE68004 before correction (**B**) and after correction (**C**). **D**, **E** Boxplot of dataset GSE73461 before correction (**D**) and after correction (**E**). KD samples are shown in blue, and control samples are shown in yellow

**Fig. 2:**
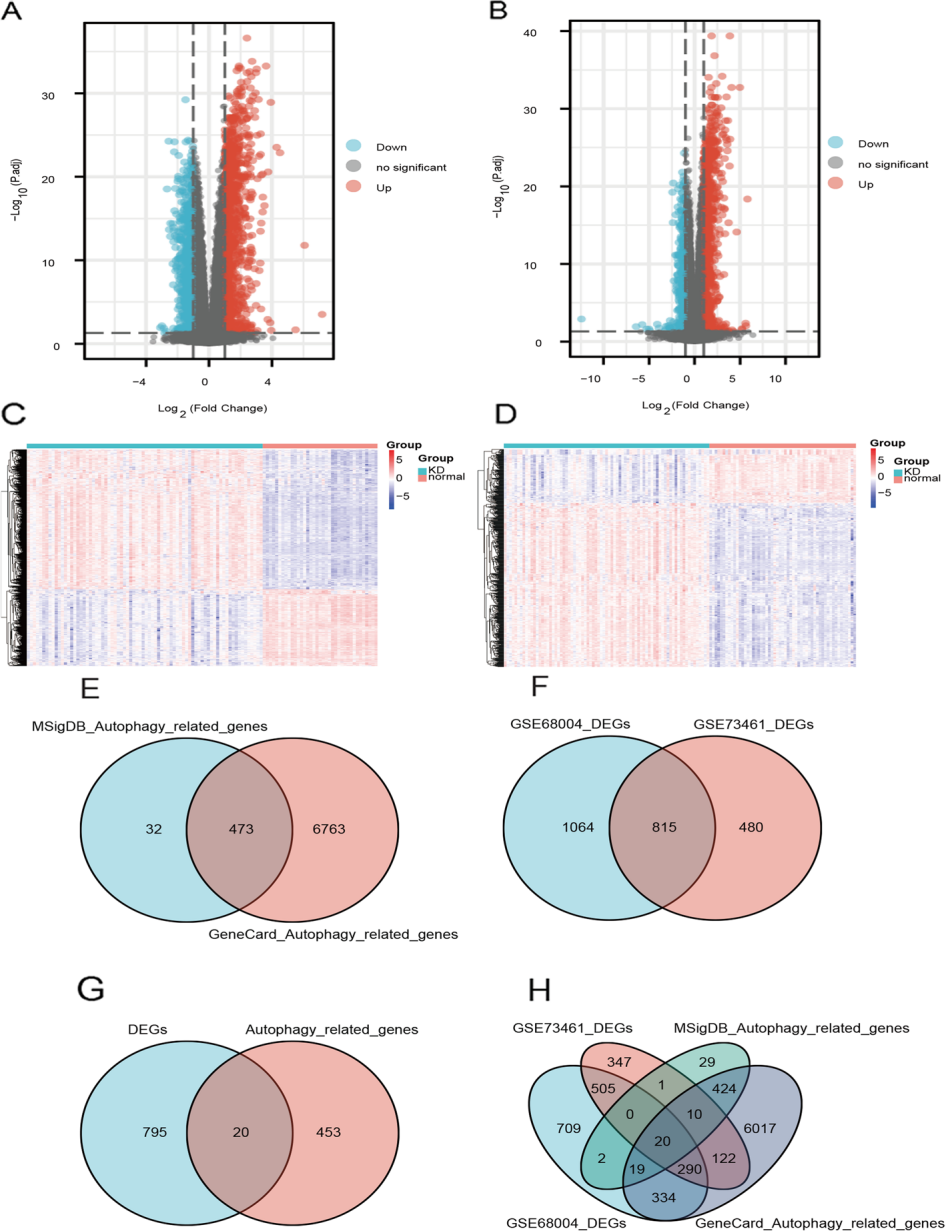
Differential expression and Venn diagram. **A**, **B** Volcano plot showing the differential expression analysis results for datasets GSE68004 (**A**) and GSE73461 (**B**). **C** Heatmap showing the differential expression analysis results for datasets GSE68004 (**C**) and GSE73461 (**D**). **E** Venn diagram showing the numbers of ARGs obtained from the MSigDB database, the GeneCards database, and both. **F** Venn diagram showing the numbers of DEGs in dataset GSE68004, dataset GSE73461, and those in both. **G** Venn diagram showing the numbers of genes that were differentially expressed in both datasets, ARGs that were obtained from both databases, and their intersection, i.e., ARGs in both databases that were differentially expressed in both datasets. **H** Venn diagram showing the numbers of DEGs of dataset GSE68004, DEGs of dataset GSE73461, ARGs in the MSigDB dataset, and ARGs in the GeneCards database

A flowchart of the present study is shown in Fig. 1A. Before data analysis, we performed corrections on the datasets GSE68004 and GSE73461 (Fig. 1B, C, D, E) to make the expression levels consistent. We performed differential expression analysis between the KD and control groups using the R package limma. In dataset GSE68004, 1879 DEGs were identified, comprising 1020 upregulated genes and 895 downregulated genes (Fig. 2A, C). In dataset GSE73461, 1295 DEGs were identified, comprising 835 upregulated genes and 460 downregulated genes (Fig. 2B, D). We generated volcano plots and heatmaps to visualize the results.

To identify genes related to autophagy, we searched the GeneCards and MSigDB databases with “autophagy” as the search keyword and downloaded the list of the autophagy-associated genes. We intersected ARGs obtained from the GeneCards and MSigDB databases and obtained 473 ARGs (Fig. 2E, Supplementary Table 2).

We intersected the DEGs in datasets GSE68004 and GSE73461 to obtain 815 DEGs (Fig. 2F). We then intersected the DEGs in both datasets with ARGs, and obtained the following 20 genes associated with cellular autophagy in KD: TSPO, SH3GLB1, FBXL2, LRRK2, WIPI1, GBA, CAMKK2, C9orf72, QSOX1, RALB, KLHL3, PIK3CB, ATP6V0E2, DRAM1, EPAS1, ATP6V1C1, GNAI3, DEPP1, WDFY3, and PLEKHF1 (Fig. 2G, H, Supplementary Table 3).

### Functional enrichment analysis of ARGs

**Fig. 3:**
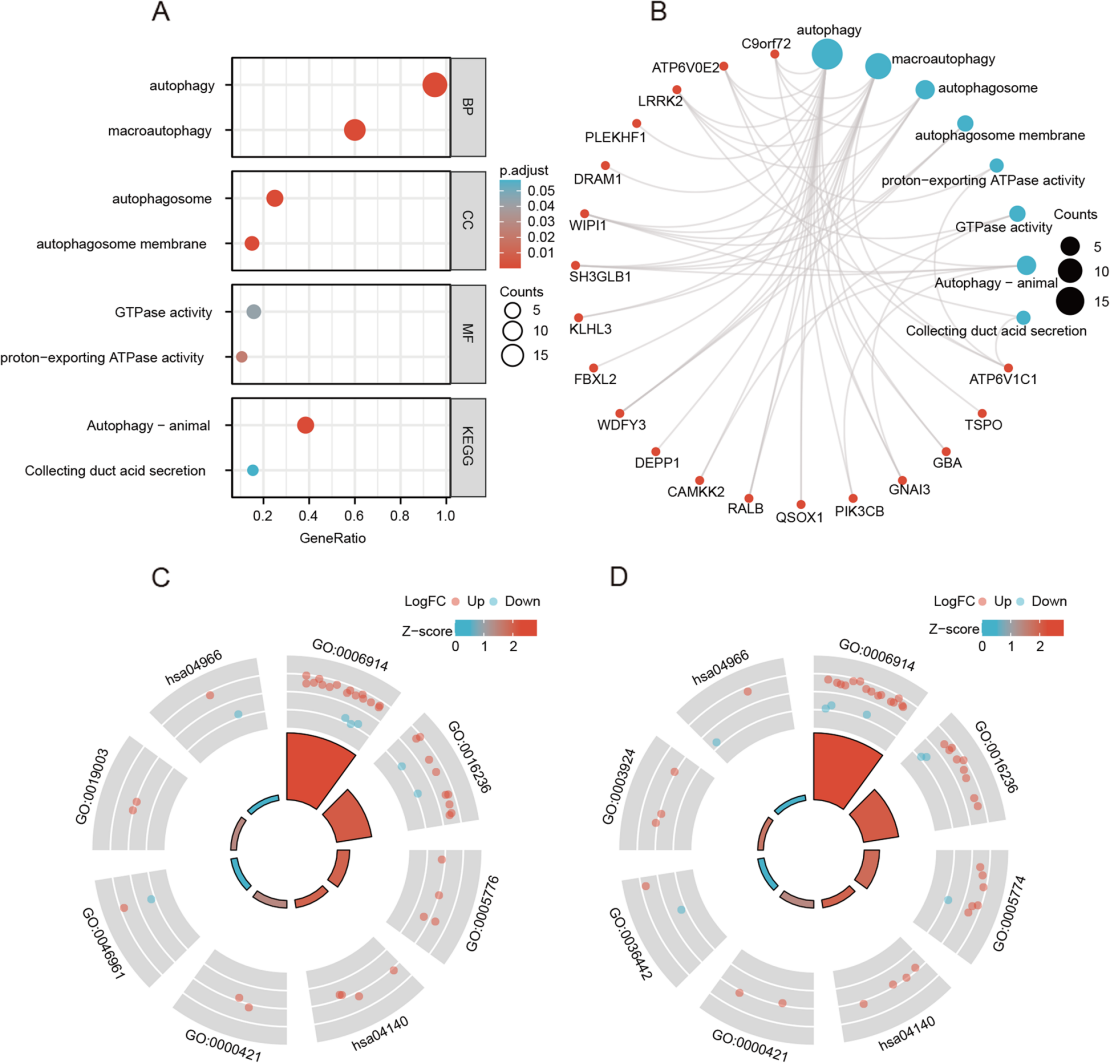
Functional enrichment analysis of ARGs in KD. **A** GO and KEGG enrichment bubble maps of ARGs in KD. **B** GO and KEGG enrichment circle maps of ARGs in KD. **C** Functional enrichment Circos maps of the GSE68004 dataset combined with log(fold change) values. **D** Functional enrichment Circos maps of the GSE73461 dataset combined with log(fold change) values

We performed GO enrichment analysis of ARGs in KD. In the biological process category, ARGs were enriched in processes such as autophagy and macro-autophagy. In the cellular component category, ARGs were enriched in components such as the vacuolar membrane and the autophagosome membrane. In the molecular function category, ARGs were enriched in proton-exporting ATPase and GTPase activity. Next, we performed KEGG pathway enrichment analysis of ARGs in KD. ARGs in KD were enriched in autophagy—animal and the collecting duct acid secretion pathway (Fig. 3). The GO and KEGG enrichment analysis results are shown in Supplementary Table 4.

### Construction of the PPI network

**Fig. 4:**
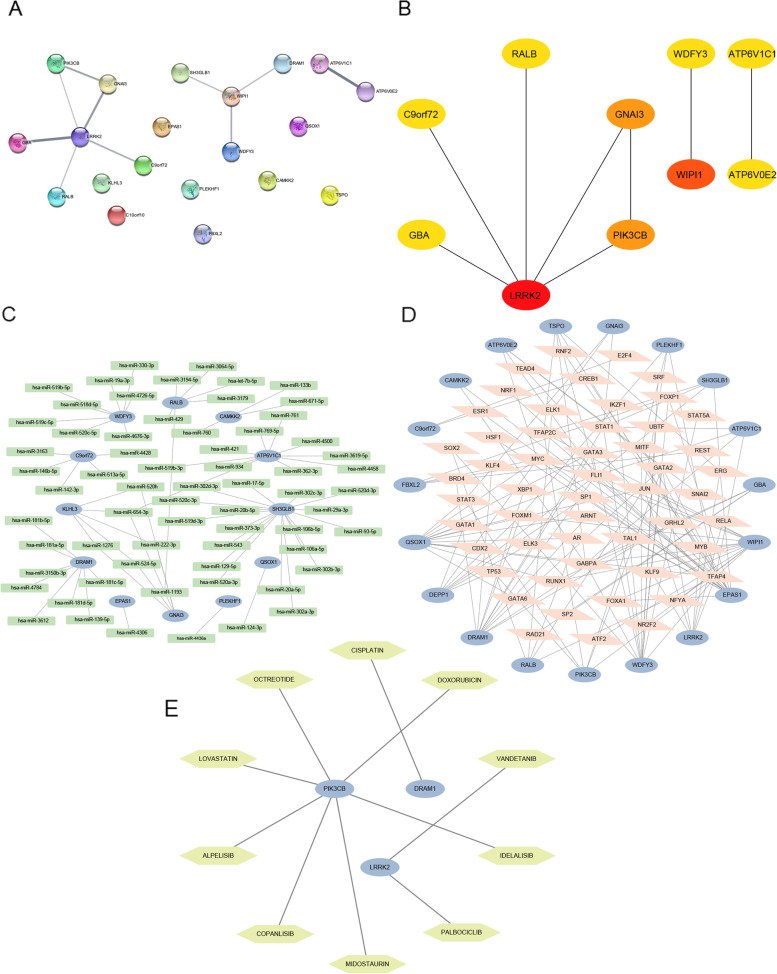
PPI network and targeting network of ARGs in KD. **A** PPI network of ARGs in KD based on the STRING database. **B** Top 10 key genes of the PPI network. **C** Network of ARGs and targeted miRNAs. **D** Network of ARGs and targeted TFs. **E** Network of ARGs and targeted drugs. Blue oval dots represent ARGs; green rounded rectangles represent miRNAs targeted by ARGs; orange quadrangles represent TFs targeted by ARGs; and yellow hexagons represent gene-related drugs

We constructed a PPI network behind the ARGs in KD based on the STRING database, which contains a total of 12 nodes and 10 edges (Fig. 4A). We used Cytoscape software to visualize the obtained PPI network, removed the non-interacting proteins, and filtered the top 10 hub genes in the network by the cytoHubba plugin (Fig. 4B). From the PPI network diagram, we can see that among the 20 ARGs, TSPO, QSOX1, PLEKHF1, FBXL2, CAMKK2, KLHL3, EPAS1, and DEPP1 are not associated with other genes, and the genes WIPI1, WDFY3, ATP6V0E2, RALB, ATP6V1C1, GBA, C9orf72, LRRK2, GNAI3, and PIK3CB had the strongest protein interactions. The protein interactions among ARGs in KD are shown in Supplementary Table 5.

### Construction of mRNA–miRNA and mRNA–TF network

To comprehensively and accurately mine the regulatory relationship between mRNA and miRNA and between mRNA and TFs in KD, we predicted the target miRNAs of ARGs in KD using the miRWalk, miRDB, miRTarBase, and starBase databases. miRNAs identified in all four databases were considered to be miRNA targets of ARGs in KD. The target TFs of ARGs in KD were predicted using the hTFtarget and knockTF databases, and TF identified in both databases were considered to be TF targets of ARGs in KD. We constructed miRNA–mRNA and mRNA–TF interaction networks using Cytoscape.

From the mRNA–miRNA network, a total of 72 miRNAs were predicted to be targeted by 12 ARGs in KD, and eight genes (GBA, TSPO, WIPI1, ATP6V0E2, LRRK2, PIK3CB, FBXL2, and DEPP1) were not predicted to target miRNAs (Fig. 4C). From the mRNA–TF network diagram, we can see that 130 TFs were predicted to be targeted by 19 ARGs in KD, and the gene KLHL3 was not predicted to target any TFs (Fig. 4D). The interaction relationships between ARGs and miRNAs and TFs in KD are shown in Supplementary Tables 6 and 7.

### Construction of a target gene–drug network

We predicted drugs that may interact with ARGs in KD based on the DGIdb database. We obtained 10 gene–drug pairs including three genes (DRAM1, LRRK2, and PIK3CB) and 10 drugs (cisplatin, vandetanib, palbociclib, copanlisib, midostaurin, octreotide, idelalisib, doxorubicin, lovastatin, and alpelisib). We then constructed a gene–drug interaction network using Cytoscape software (Fig. 4E). Among 20 ARGs, 17 genes (SH3GLB1, GBA, RALB, GNAI3, TSPO, WIPI1, QSOX1, ATP6V0E2, ATP6V1C1, C9orf72, PLEKHF1, WDFY3, FBXL2, CAMKK2, KLHL3, EPAS1, and DEPP1) were not predicted to interact with drugs. The interactions between ARGs and drugs in KD are shown in Supplementary Table 8.

### Diagnostic significance of ARGs in KD

**Fig. 5:**
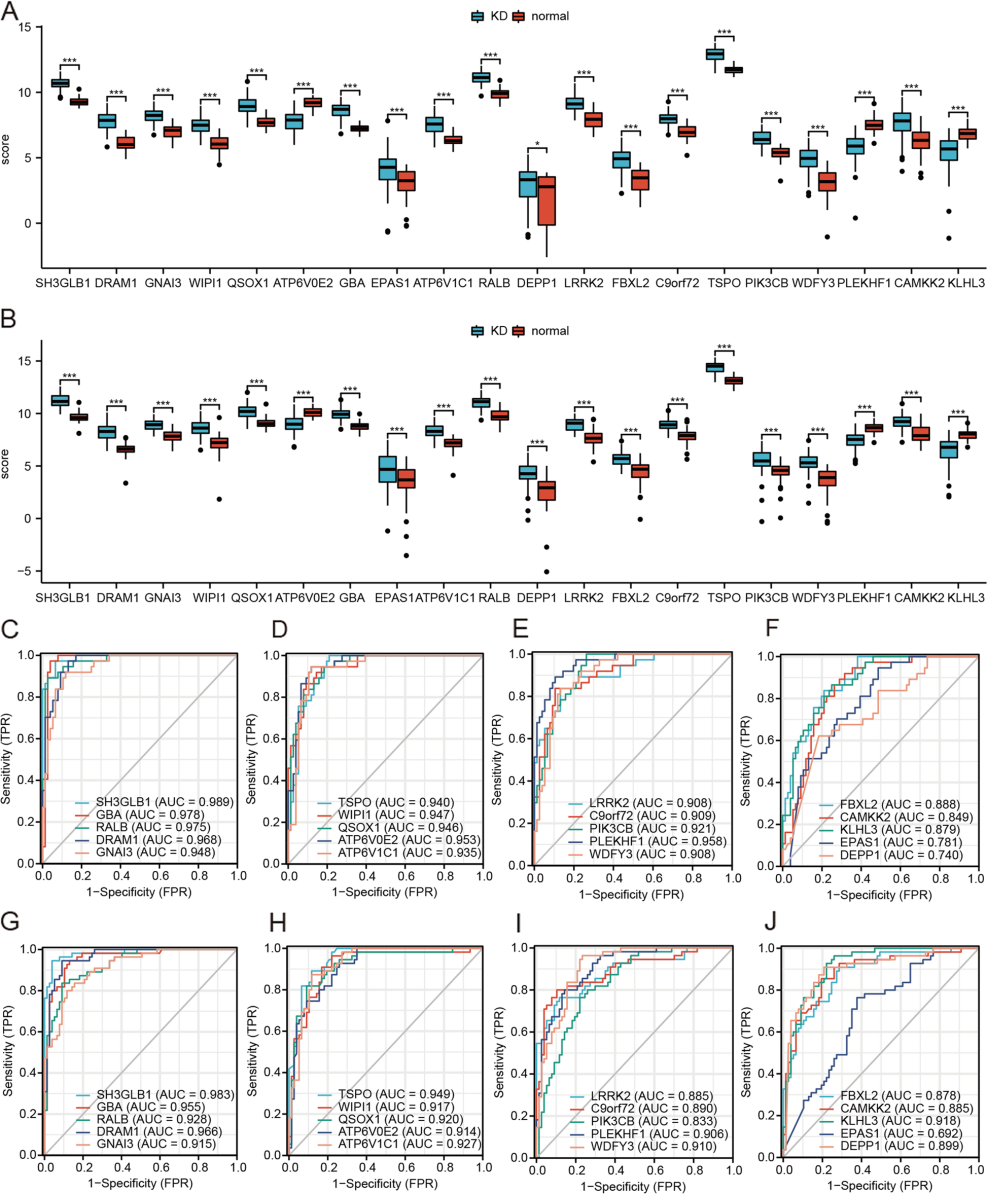
Diagnostic significance of ARGs in KD. **A**, **B** Boxplots of differential expression of ARGs in datasets GSE68004 (**A**) and GSE73461 (**B**). **C**–**F** ROC curves for genes SH3GLB1, GBA, RALB, DRAM1, and GNAI3 (**C**); TSPO, WIPI1, QSOX1, ATP6V0E2, and ATP6V1C1 (**D**); LRRK2, C9orf72, PIK3CB, PLEKHF1, and WDFY3 (**E**); and FBXL2, CAMKK2, KLHL3, EPAS1, and DEPP1 (**F**) in dataset GSE68004. (**G**–**J**). ROC curves for genes SH3GLB1, GBA, RALB, DRAM1, and GNAI3 (**G**); TSPO, WIPI1, QSOX1, ATP6V0E2, and ATP6V1C1 (**H**); LRRK2, C9orf72, PIK3CB, PLEKHF1, and WDFY3 (**I**); and FBXL2, CAMKK2, KLHL3, EPAS1, and DEPP1 (**J**) in dataset GSE73461. *P < 0.05, *P < 0.01, *P < 0.001

To determine the diagnostic value of ARGs, we plotted a ROC curve and calculated the AUC. *P*-values of < 0.05 were considered to indicate statistical significance. In dataset GSE68004, 18 genes (SH3GLB1, GBA, RALB, DRAM1, GNAI3, TSPO, WIPI1, QSOX1, ATP6V1C1, LRRK2, C9orf72, PIK3CB, EPAS1, WDFY3, FBXL2, CAMKK2, EPAS1, and DEPP1) were upregulated and two genes (ATP6V0E2 and HL3) were downregulated in KD patients (Fig. 5A). In dataset GSE73461, 17 genes (SH3GLB1, GBA, RALB, DRAM1, GNAI3, TSPO, WIPI1, QSOX1, ATP6V1C1, LRRK2, C9orf72, PIK3CB, WDFY3, FBXL2, CAMKK2, EPAS1, and DEPP1) were upregulated and three genes were downregulated in patients with KD (Fig. 5B).

From the ROC curves, we can see that in dataset GSE68004, genes SH3GLB1 (AUC = 0.989), GBA (AUC = 0.978), RALB (AUC = 0.975), DRAM1 (AUC = 0.968), GNAI3 (AUC = 0.948), TSPO (AUC = 0.94), WIPI1 (AUC = 0.947), QSOX1 (AUC = 0.946), ATP6V0E2 (AUC = 0.953), ATP6V1C1 (AUC = 0.935), LRRK2 (AUC = 0.908), C9orf72 (AUC = 0.909), PIK3CB (AUC = 0.921), PLEKHF1 (AUC = 0.958), and WDFY3 (AUC = 0.908) had a good diagnostic effect for KD patients (Fig. 5C–E). In addition, genes FBXL2 (AUC = 0.888), CAMKK2 (AUC = 0.849), KLHL3 (AUC = 0.879), EPAS1 (AUC = 0.781), and DEPP1 (AUC = 0.74) were generally effective for the diagnosis of KD patients (Fig. 5F). In dataset GSE73461, genes SH3GLB1 (AUC = 0.983), GBA (AUC = 0.955), RALB (AUC = 0.928), DRAM1 (AUC = 0.966), GNAI3 (AUC = 0.915), TSPO (AUC = 0.949), WIPI1 (AUC = 0.917), QSOX1 (AUC = 0.92), ATP6V0E2 (AUC = 0.914), ATP6V1C1 (AUC = 0.927), PIK3CB (AUC = 0.906), WDFY3 (AUC = 0.91), and KLHL3 (AUC = 0.918) had a good diagnostic effect for patients with KD (Fig. 5G–I). Genes LRRK2 (AUC = 0.885), C9orf72 (AUC = 0.89), PIK3CB (AUC = 0.833), FBXL2 (AUC = 0.878), CAMKK2 (AUC = 0.885), and DEPP1 (AUC = 0.899) were generally effective for patients with KD (Fig. 5I–J), and the diagnostic value of EPAS1 (AUC = 0.692) for KD was poor (Fig. 5J).

### Immune cell infiltration environment of ARGs

We determined the expression levels of ARGs in 22 infiltrating immune cell types using the CIBERSORT algorithm for datasets GSE68004 and GSE73461. The relationship between ARGs in KD and immune cell infiltration was investigated by Spearman analysis.

**Fig. 6:**
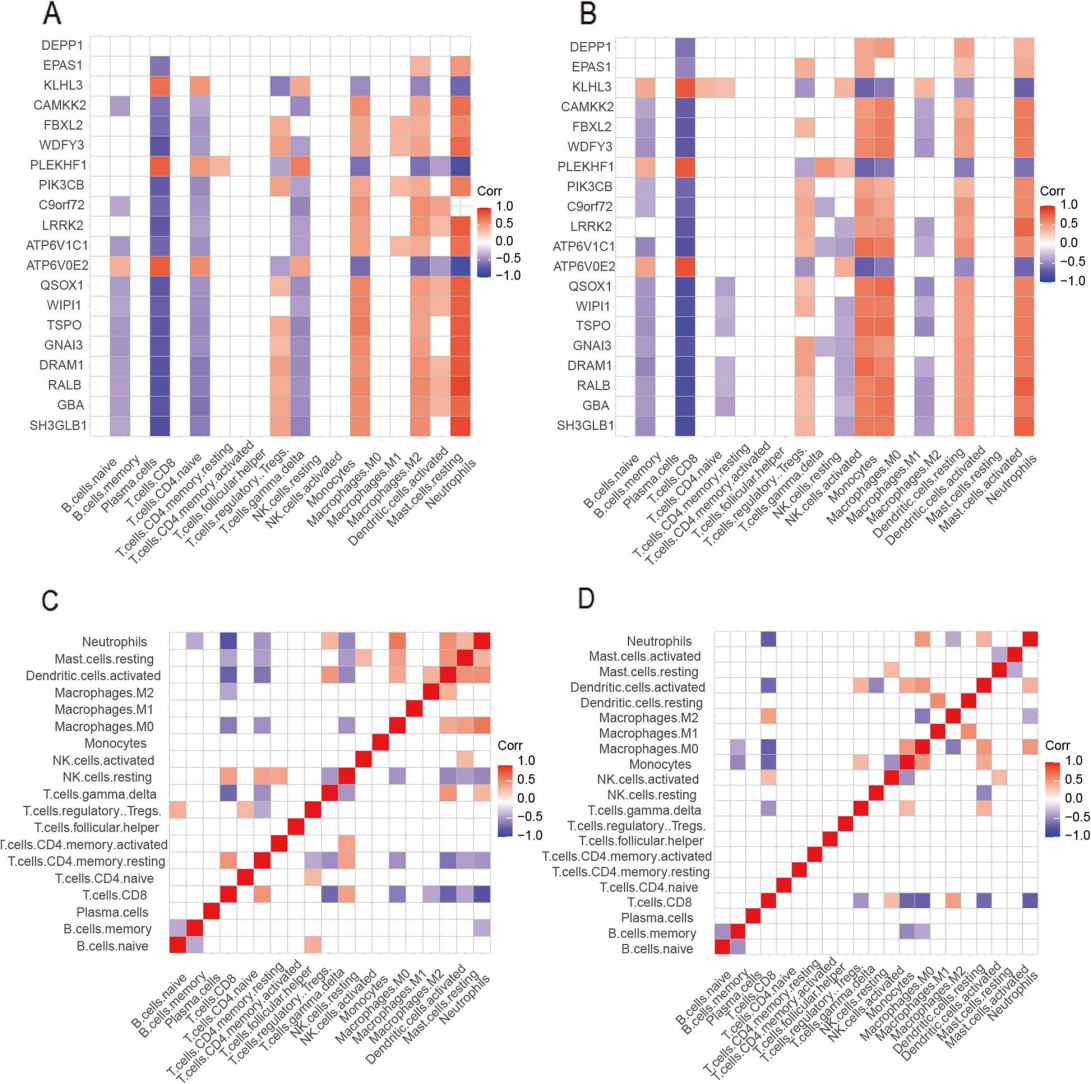
Immune cell infiltration of GSE68004 and GSE73461. **A**, **B** Heatmap of the correlation between ARGs and immune cells in KD in datasets GSE68004 and GSE73461. **C**, **D** Heatmap of the correlation between immune cells in datasets GSE68004 and GSE73461

The heatmap of the correlation between ARGs and immune cells in KD showed that in dataset GSE68004, the genes SH3GLB1 (*r* = 0.870377417, Padj = 3.37E − 24) and ATP6V0E2 (*r* =  − 0.839416377, Padj = 4.06E − 15) were negatively correlated with CD8 + T cells, and PLEKHF1 (*r* =  − 0.818109231, Padj = 1.03E − 12) was negatively correlated with neutrophils. The genes SH3GLB1 (*r* = 0.870377417, Padj = 3.37E − 24), RALB (*r* = 0.887119613, Padj = 1.11E − 26), and ATP6V0E2 (*r* = 0.821245592, Padj = 1.64E − 15) showed a positive correlation with neutrophils (Fig. 6A). In dataset GSE73461, the genes ATP6V0E2 (*r* = 0.835525466, Padj = 5.39E − 33), PLEKHF1 (*r* = 0.807322695, Padj = 5.92E − 29), and KLHL3 (*r* = 0.807131695, Padj = 6.26E − 29) showed a positive correlation with CD8 + T cells; and SH3GLB1 (*r* =  − 0.845103861, Padj = 1.52E − 34), RALB (*r* =  − 0.837560412, Padj = 2.58E − 33), and TSPO (*r* =  − 0.838906949, Padj = 1.08E − 24) showed a negative correlation with CD8 + T cells (Fig. 6B). The heatmap of the correlation between the 22 immune cell types reveals the following. In dataset GSE68004, activated dendritic cells and CD8 + T cells (*r* =  − 0.686568419, Padj = 0.010562421), resting memory CD4 + T cells (*r* =  − 0.601812691, Padj = 0.001511373), and CD8 + T cells and gamma-delta T cells (*r* =  − 0.65582577, Padj = 1.07E − 12) presented a negative correlation, whereas resting memory CD4 + T cells and CD8 + T cells (*r* = 0.531473662, Padj = 4.53E − 07), activated dendritic cells and resting mast cells (*r* = 0.535182215, Padj = 3.33E − 07), and gamma-delta T cells (*r* = 0.519659802, Padj = 1.19E − 06) presented a positive correlation (Fig. 6C). In dataset GSE73461, CD8 + T cells showed a negative correlation with monocytes (*r* =  − 0.667475424, Padj = 9.71E − 16), M0 macrophages (*r* =  − 0.698019044, Padj = 5.79E − 18), and neutrophils (*r* =  − 0.711085718, Padj = 5.31E − 19), whereas M0 macrophages showed a positive correlation with neutrophils (*r* = 0.513990197, Padj = 1.18E − 07) and activated dendritic cells (*r* = 0.514444933, Padj = 1.13E − 07), and M1 macrophages showed a positive correlation with resting dendritic cells (*r* = 0.563810017, Padj = 7.94E − 10) (Fig. 6D).

### Validation of ARG expression in KD

**Fig. 7:**
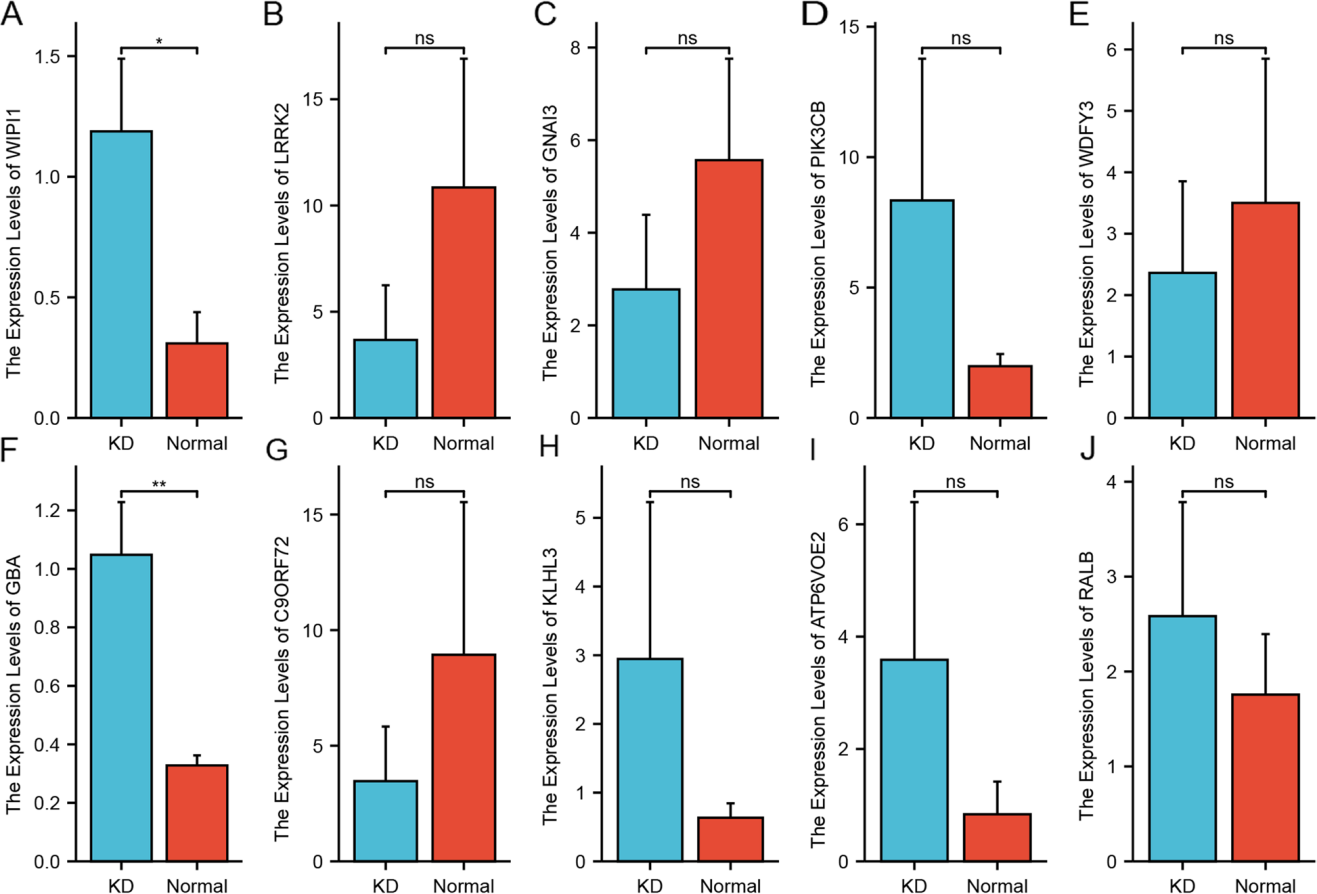
Validation of the differential expression of potential diagnostic markers, WIPI1, WDFY3, ATP6V0E2, RALB, KLHL3, GBA, C9orf72, LRRK2, GNAI3, and PIK3CB, via qRT‑PCR. *P < 0.05, **P < 0.01; ns: not significant

The expression levels of WIPI1, WDFY3, ATP6V0E2, RALB, KLHL3, GBA, C9orf72, LRRK2, GNAI3, and PIK3CB were examined by qRT-PCR in five KD blood samples and five control blood samples. Two genes showed a statistical difference (P < 0.05): WIPI1 and GBA were upregulated in KD samples compared with control samples. WDFY3, ATP6V0E2, RALB, KLHL3, C9orf72, LRRK2, GNAI3, and PIK3CB showed no significant difference between KD samples and control samples (Fig. 7).

## Discussion

KD may damage the coronary arteries. IVIG treatment reduces the incidence of coronary artery aneurysm (CAA) from 25 to 4% [29]. Some patients are at risk of CAA in adulthood despite systemic treatment [30]. Although the incidence of CAA has decreased dramatically and many treatment options are available, CAA is still not completely preventable, and it can have fatal complications [31–33]. The pathogenesis of KD is not yet clear. Genetic factors might be closely related to KD susceptibility. Genetic variants in ITPKC and CASP3 might promote excessive inflammatory responses by activating the T cell Ca2 + /NFAT signaling pathway [34, 35], and a study of cyclosporine, an inhibitor of this pathway, has been carried out in Japan in a randomized, multicenter trial demonstrating that combining cyclosporine with IVIG can reduce the incidence of CAA [36]. Moreover, the symptoms of KD are not specific, so patients are easily misdiagnosed, leading to an increased incidence of CAA. Therefore, the identification of molecular markers for early diagnosis is crucial to KD treatment and CAA prevention.

Autophagy is a conserved lysosomal degradation process that plays a key role in adaptation to metabolic stress, removal of damaged organelles, pathogen defense, and nutrient reutilization [37]. A recent study pointed out that autophagy is linked to many diseases, including cancer, inflammatory diseases, autoimmune diseases, and neurodegenerative diseases. Enhancement of autophagy/mitophagy has been shown to effectively reduce the incidence of vasculitis in KD [38]. Das et al. demonstrated that IVIG may play a role by inducing autophagy in peripheral blood mononuclear cells [39]. Resveratrol alleviates myocardial injury caused by KD by inhibiting autophagy [40]. Some studies have explored the mechanisms underlying the roles of ARGs in KD [6, 41], but no studies have investigated the mechanisms of action of ARGs in KD through bioinformatics analysis. Our bioinformatics analysis identified 20 potential ARGs associated with KD.

GO analysis revealed that these ARGs were mainly involved in biological processes such as autophagy and macro-autophagy. KEGG pathway analysis showed that most DEGs were involved in autophagy—animal. Qi et al. found that ginsenoside Rb1 may inhibit CAL inflammation by regulating the AMPK/mTOR/P70S6 autophagy signaling pathway [42].

We then established a PPI network to analyze the interaction of ARGs in KD and screened the top 10 hub genes (WIPI1, WDFY3, ATP6V0E2, RALB, ATP6V1C1, GBA, C9orf72, LRRK2, GNAI3, and PIK3CB) using Cytoscape software and the cytoHubba plugin. We also detected potential miRNAs, TFs, and drugs associated with KD, analyzed the expression levels of ARGs in tissue-infiltrating immune cells, and evaluated the diagnostic value of ARGs. These data improve our understanding of the etiology of KD.

WD repeat domain, phosphoinositide interacting 1 (WIPI1) is a component of the autophagic machinery and is activated downstream of the ULK1 and PI3 kinases to participate in autophagy. WIPI1 mRNA expression can be used as an indicator of autophagosome formation [43, 44], WIPI1 also promotes melanase transcription and melanosome formation by inhibiting TORC1, a process different from starvation-induced autophagy [45]. WD repeat and FYVE domain containing 3 (WDFY3) is essential for macro-autophagy, which is important for brain and neural development, and could also activate the TNFSF11/RANKL–TRAF6 pathway to regulate osteoclastogenesis [46, 47]. ATPase H + transporting V0 subunit e2 (ATP6V0E2) is an enzyme transporter that is widely expressed in lysosomes, and Anlotinib can activate lysosomal function. ATP6V0E2 has been identified as the key target of Anlotinib by transcriptome sequencing, and Anlotinib has been applied in the treatment of lung cancer [48]. RAS-like proto-oncogene B (RALB) is a multifunctional GTPase, and the NLRP3 inflammasome promotes RALB activation and autophagosome formation. In turn, autophagy limits interleukin-1β (IL-1β) production [49]. Lee et al. showed that the inflammatory cytokine IL-1β is related to the development of KD in a mouse model [50]. Glucosylceramidase beta 1 (GBA) encodes a lysosomal membrane protein. Homozygous mutations in GBA lead to Gaucher disease, and heterozygous GBA mutations lead to Parkinson's disease by inhibiting autophagy [51]. C9orf72-SMCR8 complex subunit (C9orf72) hexanucleotide expansion is the most common genetic cause of familial amyotrophic lateral sclerosis (ALS), and IL-1β levels are increased in the cerebrospinal fluid of ALS patients [52]. In KD, IL-1β decreases the anti-inflammatory effects of IVIG and thus increases drug resistance through activation of the TFs C/EBPβ and C/EBPδ [53]. We speculate that C9orf72 might be related to the occurrence of KD, but this hypothesis requires experimental verification. Leucine-rich repeat kinase 2 (LRRK2) can participate in inflammatory responses in vivo through the MAPK and NF-κB signaling pathways [54], Zhou et al. found that IVIG may reduce CAL development in KD by inhibiting NF-κB and p38 MAPK activation [55]. Downregulation of the LRRK2 gene in primary microglia or inhibition of its kinase activity reduced the production of tumor necrosis factor-alpha (TNFα) and IL-1β [10, 18]. G protein subunit alpha I3 (GNAI3) specifically regulates neutrophil chemotaxis through PI3Kγ signaling [56], and neutrophils may mediate vascular endothelial injury, which is the main cause of CAL secondary to KD. Integrin αIIbβ3 mediates atherothrombosis, and the key role of phosphatidylinositol-4,5-bisphosphate 3-kinase catalytic subunit beta (PIK3CB) in regulating the formation and stability of adhesion bonds of integrin αIIbβ3 may be a new target for future antithrombotic therapy [57]. In atherosclerosis, inhibition of the PI3K/Akt/mTOR pathway by microRNA-126 attenuates endothelial cell injury [58].

MiRNAs are endogenous small, non-coding RNAs. We constructed an mRNA–miRNA network and predicted a total of 72 target miRNAs, some of which have been validated. hsa-miR-19a-3p was suggested by Jone et al. to be a possible biomarker to distinguish KD from other infectious diseases [59]. Another study showed that hsa-miR-222-3p is useful for the early diagnosis of KD [60]. We further constructed TF–mRNA networks and predicted a total of 130 target TFs.

In a rat study, octreotide was shown to exert significant anti-inflammatory effects by reducing the levels of inflammatory cytokines such as TNF-α [61], and another rat study showed that octreotide inhibited the renal inflammatory response after hepatic ischemia and reperfusion injury (HIR) through the autophagic pathway [62]. PI3K inhibitors, such as idelalisib, copanlisib, and alpelisib, are used for the treatment of tumors; in addition, they may have prospects in the treatment of autoimmune diseases, inflammatory diseases, and cardiovascular diseases [63]. Several experimental and clinical studies have shown that statins such as lovastatin also exert anti-inflammatory and immunomodulatory effects [64].

We used the CIBERSORT package to evaluate the types of infiltrated immune cells in KD patients and controls and the correlation with ARG expression. Our analysis showed that SH3GLB1, ATP6V0E2, PLEKHF1, RALB, KLHL3, and TSPO were closely related to CD8 + T cells and neutrophils. A previous study showed that neutrophil counts increased rapidly in the acute phase of KD and decreased significantly in the recovery phase [65], and a mouse study showed that CD8 + T cells played a key role in KD vasculitis [66]. A heatmap of immune cell correlation showed that resting memory CD4 + T cells and CD8 + T cells were closely related to gamma-delta T cells and that CD8 + T cells were closely related to monocytes, M0 macrophages, and neutrophils. The underlying mechanisms require further investigation.

We performed ROC curve analysis of autophagy genes in KD to further verify their diagnostic value in KD. In dataset GSE68004, the genes SH3GLB1, GBA, RALB, DRAM1, GNAI3, TSPO, WIPI1, QSOX1, ATP6V0E2, ATP6V1C1, LRRK2, C9orf72, PIK3CB, PIK3CB, and WDFY3 had a good diagnostic effect for KD. In dataset GSE73461, the genes SH3GLB1, GBA, RALB, DRAM1, GNAI3, TSPO, WIPI1, QSOX1, ATP6V0E2, ATP6V1C1, PIK3CB, WDFY3, and KLHL3 had a good diagnostic effect for KD. We finally examined the expression levels of 10 genes by qRT-PCR. The expression levels of WIPI1 and GBA were consistent with our bioinformatics analysis results.

There are still some limitations in our study. First, the prevalence of KD varies by race, but KD samples in the GSE dataset are limited to KD samples from Europe and the United States. Second, the small sample size verified via qRT-PCR may reduce the confidence of the study. We will expand the sample size in future studies. Finally, the prognostic power of the identified ARGs remains unclear.

## Conclusion

A total of 20 ARGs that may be related to KD development were identified by bioinformatics analysis. WIPI1 and GBA may be potential ARGs of KD and should be further verified.

## Supplementary Information

**Additional file 1: Supplementary Table 1.** Primer sequences for quantitative real-time PCR.

**Additional file 2: Supplementary Table 2.** Autophagy-related genes.

**Additional file 3: Supplementary Table 3.** Autophagy-related genes in Kawasaki disease.

**Additional file 4: Supplementary Table 4.** GO and KEGG enrichment analyses.

**Additional file 5: Supplementary Table 5.** Protein–protein interaction network.

**Additional file 6: Supplementary Table 6.** mRNA–miRNA interaction network.

**Additional file 7: Supplementary Table 7.** mRNA–TF interaction network.

**Additional file 8: Supplementary Table 8.** mRNA–drug network.

## Data Availability

The datasets used in this study can be acquired online in repositories. The names of the repository/repositories and the accession number(s) can be found in the article.

## Abbreviations

KD: Kawasaki disease
CAD: Coronary artery disease
ARGs: Autophagy-related genes
GO: Gene Ontology
KEGG: Kyoto Encyclopedia of Genes and Genomes
PPI: Protein–protein interaction
DEGs: Differentially expressed genes
TFs: Transcription factors
MsigDB: Molecular Signatures Database
DGIdb: Drug–Gene Interaction Database
ROC: Receiver operating characteristic
qRT-PCR: Quantitative real-time PCR
miRNAs: MicroRNAs
TSPO: Translocator protein
SH3GLB1: SH3 domain containing GRB2 like, endophilin B1
FBXL2: F-box and leucine-rich repeat protein 2
LRRK2: Leucine-rich repeat kinase 2
WIPI1: WD repeat domain, phosphoinositide interacting 1
GBA: Glucosylceramidase beta 1
CAMKK2: Calcium/calmodulin-dependent protein kinase kinase 2
C9orf72: C9orf72-SMCR8 complex subunit
QSOX1: Quiescin sulfhydryl oxidase 1
RALB: RAS-like proto-oncogene B
KLHL3: Kelch-like family member 3
PIK3CB: Phosphatidylinositol-4,5-bisphosphate 3-kinase catalytic subunit beta
ATP6V0E2: ATPase H + transporting V0 subunit E2
DRAM1: DNA damage-regulated autophagy modulator 1
EPAS1: Endothelial PAS domain protein 1
PLEKHF1: Pleckstrin homology and FYVE domain containing 1
ATP6V1C1: ATPase H + transporting V1 subunit C1
GNAI3: G protein subunit alpha I3
DEPP1: DEPP autophagy regulator 1
WDFY3: WD repeat and FYVE domain containing 3

## References

[1] Esmaeili S, Mehrgou A, Kakavandi N, Rahmati Y (2020). Exploring Kawasaki disease-specific hub genes revealing a striking similarity of expression profile to bacterial infections using weighted gene co-expression network analysis (WGCNA) and co-expression modules identification tool (CEMiTool): An integrated bioinformatics and experimental study. Immunobiology.

[2] He L, Sheng Y, Huang C, Huang G (2016). Identification of Differentially Expressed Genes in Kawasaki Disease Patients as Potential Biomarkers for IVIG Sensitivity by Bioinformatics Analysis. Pediatr Cardiol.

[3] Jing F, Weng H, Pei Q, Zhang J, Liu R, Yi Q (2022). Association between serum miR-221–3p and intravenous immunoglobulin resistance in children with Kawasaki disease. Clin Exp Med.

[4] Klionsky D, Cregg J, Dunn W, Emr S, Sakai Y, Sandoval I, Sibirny A, Subramani S, Thumm M, Veenhuis M (2003). A unified nomenclature for yeast autophagy-related genes. Dev Cell.

[5] Bian M, Wang W, Song C, Pan L, Wu Y, Chen L (2022). Autophagy-Related Genes Predict the Progression of Periodontitis Through the ceRNA Network. J Inflamm Res.

[6] Qin J, Zheng Y, Ding Y, Huang C, Hou M, Li M, Qian G, Lv H (2021). Co-culture of peripheral blood mononuclear cell (PBMC) and human coronary artery endothelial cell (HCAEC) reveals the important role of autophagy implicated in Kawasaki disease. Translational pediatrics.

[7] Zheng YZ, Huang SH, Zhang JL, Hou J, Wu F, Wang WJ, Han X, Gui YH. Melatonin alleviates vascular endothelial cell damage by regulating an autophagy-apoptosis axis in Kawasaki disease. Cell Prolif. 2022;55(6):e13251. 10.1111/cpr.13251 PMC9201377 35582751

[8] Jaggi P, Mejias A, Xu Z, Yin H, Moore-Clingenpeel M, Smith B, Burns JC, Tremoulet AH, Jordan-Villegas A, Chaussabel D (2018). Whole blood transcriptional profiles as a prognostic tool in complete and incomplete Kawasaki Disease. PLoS ONE.

[9] Wright VJ, Herberg JA, Kaforou M, Shimizu C, Eleftherohorinou H, Shailes H, Barendregt AM, Menikou S, Gormley S, Berk M (2018). Diagnosis of Kawasaki Disease Using a Minimal Whole-Blood Gene Expression Signature. JAMA Pediatr.

[10] Barrett T, Wilhite SE, Ledoux P, Evangelista C, Kim IF, Tomashevsky M, Marshall KA, Phillippy KH, Sherman PM, Holko M (2013). NCBI GEO: archive for functional genomics data sets–update. Nucleic Acids Res..

[11] Davis S, Meltzer PS (2007). GEOquery: a bridge between the Gene Expression Omnibus (GEO) and BioConductor. Bioinformatics.

[12] Ritchie ME, Phipson B, Wu D, Hu Y, Law CW, Shi W, Smyth GK (2015). limma powers differential expression analyses for RNA-sequencing and microarray studies. Nucleic Acids Res.

[13] Stelzer G, Rosen N, Plaschkes I, Zimmerman S, Twik M, Fishilevich S, Stein TI, Nudel R, Lieder I, Mazor Y (2016). The GeneCards Suite: From Gene Data Mining to Disease Genome Sequence Analyses. Curr Protoc Bioinformatics..

[14] Liberzon A, Birger C, Thorvaldsdóttir H, Ghandi M, Mesirov JP, Tamayo P (2015). The Molecular Signatures Database (MSigDB) hallmark gene set collection. Cell Syst.

[15] Gene Ontology Consortium. Gene Ontology Consortium: going forward. Nucleic Acids Res. 2015;43(Database issue):D1049–56. 10.1093/nar/gku1179 PMC4383973 25428369

[16] Ogata H, Goto S, Sato K, Fujibuchi W, Bono H, Kanehisa M (1999). KEGG: Kyoto Encyclopedia of Genes and Genomes. Nucleic Acids Res.

[17] Yu G, Wang LG, Han Y, He QY (2012). clusterProfiler: an R package for comparing biological themes among gene clusters. OMICS.

[18] Szklarczyk D, Gable AL, Nastou KC, Lyon D, Kirsch R, Pyysalo S, Doncheva NT, Legeay M, Fang T, Bork P (2021). The STRING database in 2021: customizable protein-protein networks, and functional characterization of user-uploaded gene/measurement sets. Nucleic Acids Res.

[19] Shannon P, Markiel A, Ozier O, Baliga NS, Wang JT, Ramage D, Amin N, Schwikowski B, Ideker T (2003). Cytoscape: a software environment for integrated models of biomolecular interaction networks. Genome Res.

[20] Chin CH, Chen SH, Wu HH, Ho CW, Ko MT, Lin CY (2014). cytoHubba: identifying hub objects and sub-networks from complex interactome. BMC Syst Biol.

[21] Dweep H, Gretz N, Sticht C (2014). miRWalk database for miRNA-target interactions. Methods Mol Biol.

[22] Chen Y, Wang X (2020). miRDB: an online database for prediction of functional microRNA targets. Nucleic Acids Res.

[23] Huang HY, Lin YC, Li J, Huang KY, Shrestha S, Hong HC, Tang Y, Chen YG, Jin CN, Yu Y (2020). miRTarBase 2020: updates to the experimentally validated microRNA-target interaction database. Nucleic Acids Res.

[24] Li JH, Liu S, Zhou H, Qu LH, Yang JH (2014). starBase v2.0: decoding miRNA-ceRNA, miRNA-ncRNA and protein-RNA interaction networks from large-scale CLIP-Seq data. Nucleic Acids Res..

[25] Zhang Q, Liu W, Zhang HM, Xie GY, Miao YR, Xia M, Guo AY (2020). hTFtarget: A Comprehensive Database for Regulations of Human Transcription Factors and Their Targets. Genomics Proteomics Bioinformatics.

[26] Cotto KC, Wagner AH, Feng YY, Kiwala S, Coffman AC, Spies G, Wollam A, Spies NC, Griffith OL, Griffith M (2018). DGIdb 30: a redesign and expansion of the drug-gene interaction database. Nucleic Acids Res..

[27] Chen B, Khodadoust MS, Liu CL, Newman AM, Alizadeh AA (2018). Profiling Tumor Infiltrating Immune Cells with CIBERSORT. Methods Mol Biol.

[28] Robin X, Turck N, Hainard A, Tiberti N, Lisacek F, Sanchez JC, Müller M (2011). pROC: an open-source package for R and S+ to analyze and compare ROC curves. BMC Bioinformatics.

[29] Grasa CD, Fernandez-Cooke E, Sánchez-Manubens J, Antón J, Crespo D, García M, López A, Lirola Cruz MJ, Díaz-Delgado de la Peña R, Calvo C (2019). Kawasaki disease in infants 3 months of age and younger: a multicentre Spanish study. Ann Rheum Dis.

[30] Matsuura H, Ohya M (2018). Coronary-Artery Occlusion from Kawasaki's Disease. N Engl J Med.

[31] Newburger J, Sleeper L, McCrindle B, Minich L, Gersony W, Vetter V, Atz A, Li J, Takahashi M, Baker A (2007). Randomized trial of pulsed corticosteroid therapy for primary treatment of Kawasaki disease. N Engl J Med.

[32] Tremoulet AH, Jain S, Jaggi P, Jimenez-Fernandez S, Pancheri JM, Sun X, Kanegaye JT, Kovalchin JP, Printz BF, Ramilo O (2014). Infliximab for intensification of primary therapy for Kawasaki disease: a phase 3 randomised, double-blind, placebo-controlled trial. The Lancet.

[33] Kobayashi T, Saji T, Otani T, Takeuchi K, Nakamura T, Arakawa H, Kato T, Hara T, Hamaoka K, Ogawa S (2012). Efficacy of immunoglobulin plus prednisolone for prevention of coronary artery abnormalities in severe Kawasaki disease (RAISE study): a randomised, open-label, blinded-endpoints trial. The Lancet.

[34] Onouchi Y, Gunji T, Burns J, Shimizu C, Newburger J, Yashiro M, Nakamura Y, Yanagawa H, Wakui K, Fukushima Y (2008). ITPKC functional polymorphism associated with Kawasaki disease susceptibility and formation of coronary artery aneurysms.

[35] Onouchi Y, Ozaki K, Buns J, Shimizu C, Hamada H, Honda T, Terai M, Honda A, Takeuchi T, Shibuta S (2010). Common variants in CASP3 confer susceptibility to Kawasaki disease. Nat Genet.

[36] Hamada H, Suzuki H, Onouchi Y, Ebata R, Terai M, Fuse S, Okajima Y, Kurotobi S, Hirai K, Soga T (2019). Efficacy of primary treatment with immunoglobulin plus ciclosporin for prevention of coronary artery abnormalities in patients with Kawasaki disease predicted to be at increased risk of non-response to intravenous immunoglobulin (KAICA): a randomised controlled, open-label, blinded-endpoints, phase 3 trial. Lancet (London, England).

[37] Levine B, Kroemer G (2019). Biological Functions of Autophagy Genes: A Disease Perspective. Cell.

[38] Marek-Iannucci S, Ozdemir AB, Moreira D, Gomez AC, Lane M, Porritt RA, Lee Y, Shimada K, Abe M, Stotland A (2021). Autophagy-mitophagy induction attenuates cardiovascular inflammation in a murine model of Kawasaki disease vasculitis. JCI insight..

[39] Das M, Karnam A, Stephen-Victor E, Gilardin L, Bhatt B, Kumar Sharma V, Rambabu N, Patil V, Lecerf M, Kasermann F (2020). Intravenous immunoglobulin mediates anti-inflammatory effects in peripheral blood mononuclear cells by inducing autophagy. Cell Death Dis.

[40] Xiong F, Liu R, Guo H, Wu D, Sun N (2021). Resveratrol alleviates Kawasaki disease-induced myocardial injury via inhibition of apoptosis and autophagy. Zhong Nan Da Xue Xue Bao Yi Xue Ban.

[41] Huang FC, Huang YH, Kuo HC, Li SC (2020). Identifying Downregulation of Autophagy Markers in Kawasaki Disease. Children (Basel, Switzerland)..

[42] Qi SH, Xiao F, Wei B, Qin C (2020). Value of ginsenoside Rb1 in alleviating coronary artery lesion in a mouse model of Kawasaki disease. Zhongguo dang dai er ke za zhi = Chin J Contemp Pediatr.

[43] Bakula D, Müller A, Zuleger T, Takacs Z, Franz-Wachtel M, Thost A, Brigger D, Tschan M, Frickey T, Robenek H (2017). WIPI3 and WIPI4 β-propellers are scaffolds for LKB1-AMPK-TSC signalling circuits in the control of autophagy. Nat Commun.

[44] Tsuyuki S, Takabayashi M, Kawazu M, Kudo K, Watanabe A, Nagata Y, Kusama Y, Yoshida K (2014). Detection of WIPI1 mRNA as an indicator of autophagosome formation. Autophagy.

[45] Ho H, Kapadia R, Al-Tahan S, Ahmad S, Ganesan AJT (2011). WIPI1 coordinates melanogenic gene transcription and melanosome formation via TORC1 inhibition. J Biol Chem.

[46] Isakson P, Lystad A, Breen K, Koster G, Stenmark H, Simonsen A (2013). TRAF6 mediates ubiquitination of KIF23/MKLP1 and is required for midbody ring degradation by selective autophagy. Autophagy.

[47] Kadir R, Harel T, Markus B, Perez Y, Bakhrat A, Cohen I, Volodarsky M, Feintsein-Linial M, Chervinski E, Zlotogora J (2016). ALFY-Controlled DVL3 Autophagy Regulates Wnt Signaling. Determining Human Brain Size.

[48] Sun X, Shu Y, Yan P, Huang H, Gao R, Xu M, Lu L, Tian J, Huang D, Zhang J (2020). Transcriptome profiling analysis reveals that ATP6V0E2 is involved in the lysosomal activation by anlotinib. Cell Death Dis.

[49] Shi C, Shenderov K, Huang N, Kabat J, Abu-Asab M, Fitzgerald K, Sher A, Kehrl J (2012). Activation of autophagy by inflammatory signals limits IL-1β production by targeting ubiquitinated inflammasomes for destruction. Nat Immunol..

[50] Lee Y, Schulte D, Shimada K, Chen S, Crother T, Chiba N, Fishbein M, Lehman T, Arditi MJC (2012). Interleukin-1β is crucial for the induction of coronary artery inflammation in a mouse model of Kawasaki disease. Circulation.

[51] Li H, Ham A, Ma T, Kuo S, Kanter E, Kim D, Ko H, Quan Y, Sardi S, Li A (2019). Mitochondrial dysfunction and mitophagy defect triggered by heterozygous GBA mutations. Autophagy.

[52] Pinilla G, Kumar A, Floaters M, Pardo C, Rothstein J, Ilieva H (2021). Increased synthesis of pro-inflammatory cytokines in C9ORF72 patients. Amyotroph Lateral Scler Frontotemporal Degener.

[53] Inoue T, Miyashita M, Murakami S, Igarashi A, Motomura K, Abe J, Matsumoto K, Matsuda A (2020). IL-1β and IL-17A are involved in IVIG resistance through activation of C/EBPβ and δ in a coronary artery model of Kawasaki disease. Allergy.

[54] Wallings R, Tansey M (2019). LRRK2 regulation of immune-pathways and inflammatory disease. Biochem Soc Trans.

[55] Zhou C, Huang M, Xie L, Shen J, Xiao T, Wang R (2015). IVIG inhibits TNF-α-induced MMP9 expression and activity in monocytes by suppressing NF-κB and P38 MAPK activation. Int J Clin Exp Pathol..

[56] Kuwano Y, Adler M, Zhang H, Groisman A, Ley K (2016). Gαi2 and Gαi3 Differentially Regulate Arrest from Flow and Chemotaxis in Mouse Neutrophils. J Immunol..

[57] Jackson S, Schoenwaelder S, Goncalves I, Nesbitt W, Yap C, Wright C, Kenche V, Anderson K, Dopheide S, Yuan Y (2005). PI 3-kinase p110beta: a new target for antithrombotic therapy. Nat Med.

[58] Tang F, Yang T (2018). MicroRNA-126 alleviates endothelial cells injury in atherosclerosis by restoring autophagic flux via inhibiting of PI3K/Akt/mTOR pathway. Biochem Biophys Res Commun..

[59] Jone P, Korst  A, Karimpour-Fard A, Thomas T, Dominguez S, Heizer H, Anderson M, Glode M, Sucharov C, Miyamoto S (2020). Circulating microRNAs differentiate Kawasaki Disease from infectious febrile illnesses in childhood. J Mol Cell Cardiol.

[60] Wang B, Wang L, Cheng F, Lv H, Sun L, Wei D, Pu Y, Wu J, Hou Y, Wen B (2019). MiR-222–3p in Platelets Serves as a Distinguishing Marker for Early Recognition of Kawasaki Disease. Front Pediatr..

[61] Karalis K, Mastorakos G, Chrousos G, Tolis G (1994). Somatostatin analogues suppress the inflammatory reaction in vivo. J Clin Invest..

[62] Sun H, Zou S, Candiotti K, Peng Y, Zhang Q, Xiao W, Wen Y, Wu J, Yang J (2017). Octreotide Attenuates Acute Kidney Injury after Hepatic Ischemia and Reperfusion by Enhancing Autophagy. Sci Rep..

[63] Vyas P, Vohora D (2017). Phosphoinositide-3-kinases as the Novel Therapeutic Targets for the Inflammatory Diseases: Current and Future Perspectives. Curr Drug Targets..

[64] Dehnavi S, Sohrabi N, Sadeghi M, Lansberg P, Banach M, Al-Rasadi K, Johnston T (2020). Sahebkar AJP and therapeutics. Statins and autoimmunity: State-of-the-art Pharmacol Ther.

[65] Hokibara S, Kobayashi N, Kobayashi K, Shigemura T, Nagumo H, Takizawa M, Yamazaki T, Agematsu K (2016). Markedly elevated CD64 expression on neutrophils and monocytes as a biomarker for diagnosis and therapy assessment in Kawasaki disease. Inflamm Res..

[66] Noval Rivas M, Lee Y, Wakita D, Chiba N, Dagvadorj J, Shimada K, Chen S, Fishbein M, Lehman T, Crother T (2017). CD8+ T Cells Contribute to the Development of Coronary Arteritis in the Lactobacillus casei Cell Wall Extract-Induced Murine Model of Kawasaki Disease. Arthritis Rheumatol.

