# Supplementary material for: Identification and validation of autophagy-related genes in Kawasaki disease
Source: Hereditas. 2023 Apr 21;160:17. doi: 10.1186/s41065-023-00278-9 (PMC10120123; doi:10.1186/s41065-023-00278-9)
Supplement: Supplementary file 1 — Additional file 1: Supplementary Table 1. Primer sequences for quantitative real-time PCR. [file 41065_2023_278_MOESM1_ESM.docx]

**Supplementary table 1**. Primer Sequences for Quantitative real-time PCR

| Gene Names | Forward （5'-3') | Reverse (3ʹ-5ʹ) |
| --- | --- | --- |
| GAPDH | GGAAGCTTGTCATCAATGGAAATC | TGATGACCCTTTTGGCTCCC |
| WIPI1 | TTGCTTGGCTCAGGAACAACAGA | GTCCTCAGAATAACCTGGCACCG |
| RALB | GACTACGCAGCCATTCGAGATA | CGCTCCTCTAGGTCAGACTTGTT |
| GBA | TGGACCGACTGGAACCTTGC | AGCCCTCAGGAATGAACTTGC |
| WDFY3 | AATGTTTGCTGGGCTTTCTTGT | CATCTTTGGATTCTGCCTCTTT |
| LRRK2 | AGCCAATGATGAAGTCCAGAAAC | CAAGGAATCGCTAGGGAATGTA |
| GNAI3 | ATTAAACGGTTATGGCGAGATG | GTGTTTCTACAATGCCTGTGGTC |
| PIK3CB | GCTGCCTGCGACAGATGAGT | GCCCTATCCTCCGATTACCAAG |
| ATP6V0E2 | ATCTTCACCACGTTCTGGGG | AGCTGGGCACAAGAGGTAAC |
| KLHL3 | CCATGAAATACCATCTCCTCCCT | CCTCCTCGAAATCATAGCACTCC |
| C9orf72 | CCTGTGGCTGTTCCGTTGTAGTA | GCTTCACATAACCTGGAGCATTT |
