# Supplementary material for: Identification and validation of autophagy-related genes in Kawasaki disease
Source: Hereditas. 2023 Apr 21;160:17. doi: 10.1186/s41065-023-00278-9 (PMC10120123; doi:10.1186/s41065-023-00278-9)
Supplement: Supplementary file 2 — Additional file 2: Supplementary Table 2. Autophagy-related genes. [file 41065_2023_278_MOESM2_ESM.docx]

**Supplementary table 2**. Autophagy-related genes

| STUB1 | WAC | ATG12 | C9orf72 |
| --- | --- | --- | --- |
| CLU | SNX7 | ATP6V1G1 | TRIML2 |
| PLK3 | PIK3C2A | VPS26A | DHRSX |
| SYNPO2 | PIK3CB | KEAP1 | RNF152 |
| EEF1A1 | PIP4K2A | HUWE1 | ATF6 |
| EEF1A2 | TPCN1 | CDC37 | RAB3GAP1 |
| ATP13A2 | TRIM34 | HK2 | TRIM32 |
| GFAP | ATG16L1 | MUL1 | FOXO1 |
| HSPA8 | WIPI1 | ATP5IF1 | FOXO3 |
| HSP90AA1 | RNF31 | MFN2 | ATG2A |
| LAMP2 | TRIM68 | PHB2 | LARP1 |
| CTSA | VPS13D | WDR81 | UFL1 |
| SNCA | PRKAA1 | RETREG3 | SIRT1 |
| SNRNP70 | AMBRA1 | WDFY3 | SH3BP4 |
| ATG5 | PRKAA2 | ATG4B | RAB3GAP2 |
| BAG3 | PRKD1 | ARFIP2 | FBXO7 |
| SPTLC1 | MAPK3 | WIPI2 | SVIP |
| ADRB2 | BAD | KLHL3 | GPSM1 |
| HTT | ELAPOR1 | LGALS8 | HSPB8 |
| AUP1 | TP53INP2 | MAP1LC3C | SESN1 |
| RAB7A | RALB | PJVK | GNAI3 |
| SESN2 | TRIM27 | TEX264 | RUFY4 |
| SPTLC2 | SCOC | UFC1 | SLC25A4 |
| WASHC1 | ROCK1 | UFM1 | TBK1 |
| RNF41 | MOAP1 | RETREG1 | SLC25A5 |
| ATG7 | NOD2 | ATG2B | GSK3A |
| RRAGA | BNIP3 | RNF213 | GSK3B |
| EHMT2 | BNIP3L | DDRGK1 | HIF1A |
| TLK2 | TRIM21 | TAFAZZIN | HMGB1 |
| WDR6 | STK11 | UBA5 | TRIML1 |
| LRRK2 | SUPT5H | MAP1LC3B | STING1 |
| CHMP4B | MAP3K7 | ATG4D | IRGM |
| SMCR8 | UVRAG | SQSTM1 | IFNG |
| ADRA1A | VDAC1 | STBD1 | IL4 |
| DAP | PLEKHF1 | ACBD5 | KDR |
| EIF4E | TFEB | ATG13 | SNX30 |
| EIF4G1 | FYCO1 | RB1CC1 | MEFV |
| EIF4G2 | EPM2A | LRBA | PAFAH1B2 |
| AKT1 | PIP4K2C | GABARAP | PRKN |
| FOXK1 | ZC3H12A | GABARAPL2 | SH3GLB1 |
| SIRT2 | NPRL3 | ATG4A | ATP6V1G2 |
| SMG1 | TRIM8 | GABARAPL1 | ATP6V0A1 |
| TAB2 | PIP4K2B | ULK3 | VPS13C |
| SCFD1 | ULK1 | IFNA1 | WDR41 |
| CLEC16A | IKBKG | INS | FBXW7 |
| MTOR | TRIM5 | BECN2 | DRAM1 |
| TAB3 | SNX4 | ATG3 | PARL |
| POLDIP2 | RIPK2 | ATG4C | PRKACA |
| PTPN22 | LACRT | ATG10 | VPS35 |
| GATA4 | LRSAM1 | ATG101 | EXOC1 |
| NUPR1 | MTDH | DCTN1 | CISD1 |
| GOLGA2 | ORMDL3 | CFTR | MAPK8 |
| CHMP4A | TP53INP1 | VIM | PSAP |
| HGF | TMEM59 | IFT88 | GPR137 |
| HMOX1 | DEPDC5 | FUNDC1 | RPTOR |
| HTR2B | ULK2 | TSG101 | TRIB3 |
| NRBP2 | TRIM14 | DYNC1I2 | RRAGD |
| IL10 | HDAC6 | CHMP2B | SNX6 |
| IL10RA | DNM1L | DYNLL1 | RHEB |
| FOXK2 | CDK5 | HSP90AB1 | EXOC4 |
| KIF25 | ZMPSTE24 | TOMM22 | RRAGC |
| LEP | RRAGB | CSNK2A1 | BOK |
| LEPR | HAX1 | MAP1LC3A | SREBF1 |
| MIRLET7B | DEPP1 | TUBB4A | SREBF2 |
| MIR199A1 | VPS26B | PLIN3 | GPR137B |
| MAGEA3 | OSBPL7 | PRKAG2 | UCHL1 |
| MAGEA6 | RAB39B | LAMTOR3 | FZD5 |
| MCL1 | CLN3 | PRKAB1 | CAPN1 |
| MET | DRAM2 | CHMP3 | CAPNS1 |
| MT3 | SOGA1 | PRKAG3 | CASP3 |
| MTM1 | CRYBA1 | EPAS1 | KAT8 |
| NPC1 | CSNK2A2 | TUBA1B | WDR24 |
| PIK3CA | EXOC8 | TUBA4A | CDK5R1 |
| TREM2 | ATP6V0E2 | TOMM40 | MTMR3 |
| RASIP1 | DAPK3 | CHMP2A | USP13 |
| TMEM39A | DDIT3 | PRKAB2 | ATP6V0E1 |
| MTMR8 | NLRP6 | LAMTOR5 | ATP6V1E2 |
| UBQLN4 | CTTN | DYNC1LI2 | USP10 |
| TIGAR | EP300 | TUBB2A | MTMR4 |
| TBC1D14 | ERCC4 | PEX5 | ATP6V0D1 |
| USP36 | ERN1 | VPS37B | TUBA8 |
| QSOX1 | TPCN2 | WDR45B | HSF1 |
| BCL2 | MAPK15 | MVB12A | TUBB4B |
| RNF5 | ATG14 | RPS27A | NBR1 |
| PINK1 | USP33 | DYNC1LI1 | DYNC1H1 |
| MTMR9 | MTCL1 | CHMP7 | SRC |
| STAT3 | EXOC7 | PLIN2 | ATG9A |
| TSPO | ATP6V0A2 | LAMTOR1 | CSNK2B |
| TP53 | DAPK2 | UBC | UBA52 |
| TSC1 | ATP6V0D2 | TOMM70 | UBE2V1 |
| TSC2 | ATP6V1C2 | VPS37A | HBB |
| PHF23 | ABL1 | DYNC1I1 | PGAM5 |
| ZKSCAN3 | SNX32 | PCNT | TUBB3 |
| CPTP | MFSD8 | VPS28 | TUBB8 |
| USP30 | FBXL2 | MTMR14 | DYNLL2 |
| KLHL22 | TECPR1 | CHMP4C | DEPTOR |
| EIF4G3 | DCAF12 | UBAP1 | AKT1S1 |
| BECN1 | GAPDH | VCP | ZFYVE1 |
| HERC1 | GBA | TUBA1A | ATG16L2 |
| BMF | ABL2 | TUBA1C | TOMM5 |
| DAPL1 | LAMP3 | MLST8 | TUBB6 |
| SEC22B | SNX5 | VPS37C | CHMP6 |
| FEZ2 | HTRA2 | UBB | VPS37D |
| FEZ1 | PYCARD | MFN1 | UBE2N |
| KDM4A | RMC1 | TOMM20 | ATG9B |
| RUBCN | UBQLN2 | NEDD4 | PRKAG1 |
| BCL2L11 | UBQLN1 | CISD2 | WDR45 |
| OPTN | PIK3R4 | TBC1D25 | SNX18 |
| TRIM13 | HSPB1 | ATP6V1D | PARK7 |
| CALCOCO2 | ACER2 | ATP6V1H | TRIM6 |
| TRIM22 | IFI16 | VPS29 | SESN3 |
| NOD1 | ITPR1 | ATP6V1A | TICAM1 |
| TRIM38 | SOGA3 | ATP6V1B1 | DAPK1 |
| KAT5 | MAPT | ATP6V1B2 | DCN |
| NPRL2 | RAB8A | ATP6V0C | FLCN |
| CAMKK2 | ATM | ATP6V1C1 | TRIM65 |
| CERS1 | ATP6V0B | PIK3C3 | ENDOG |
| PLK2 | MID2 | ATP6V1E1 | PIK3R2 |
| PIM2 |  |  |  |
