# Supplementary material for: Identification and validation of autophagy-related genes in Kawasaki disease
Source: Hereditas. 2023 Apr 21;160:17. doi: 10.1186/s41065-023-00278-9 (PMC10120123; doi:10.1186/s41065-023-00278-9)
Supplement: Supplementary file 3 — Additional file 3: Supplementary Table 3. Autophagy-related genes in Kawasaki disease. [file 41065_2023_278_MOESM3_ESM.docx]

**Supplementary table 3**. Autophagy-related genes in kawasaki disease

| TSPO | SH3GLB1 | FBXL2 | LRRK2 |
| --- | --- | --- | --- |
| WIPI1 | GBA | CAMKK2 | C9orf72 |
| QSOX1 | RALB | KLHL3 | PIK3CB |
| ATP6V0E2 | DRAM1 | EPAS1 | PLEKHF1 |
| ATP6V1C1 | GNAI3 | DEPP1 | WDFY3 |
