# Supplementary material for: Identification and validation of autophagy-related genes in Kawasaki disease
Source: Hereditas. 2023 Apr 21;160:17. doi: 10.1186/s41065-023-00278-9 (PMC10120123; doi:10.1186/s41065-023-00278-9)
Supplement: Supplementary file 4 — Additional file 4: Supplementary Table 4. GO and KEGG enrichment analyses. [file 41065_2023_278_MOESM4_ESM.docx]

**Supplementary table 4**. GO and KEGG enrichment analysis

| ONTOLOGY | Description | pvalue | p.adjust | qvalue |
| --- | --- | --- | --- | --- |
| BP | autophagy | 1.60E-29 | 6.98E-27 | 4.28E-27 |
| BP | macroautophagy | 2.18E-17 | 4.75E-15 | 2.91E-15 |
| CC | vacuolar membrane | 3.43E-09 | 3.92E-07 | 2.39E-07 |
| CC | autophagosome membrane | 6.24E-06 | 0.000237034 | 0.000144453 |
| MF | proton-exporting ATPase activity | 0.00046661 | 0.022720574 | 0.010606401 |
| MF | GTPase activity | 0.004739638 | 0.042558359 | 0.01986706 |
| KEGG | Autophagy - animal | 1.51E-06 | 0.000209445 | 0.000160197 |
| KEGG | Collecting duct acid secretion | 0.000820785 | 0.057044574 | 0.043631215 |
