# Supplementary material for: Identification and validation of autophagy-related genes in Kawasaki disease
Source: Hereditas. 2023 Apr 21;160:17. doi: 10.1186/s41065-023-00278-9 (PMC10120123; doi:10.1186/s41065-023-00278-9)
Supplement: Supplementary file 5 — Additional file 5: Supplementary Table 5. Protein–protein interaction network. [file 41065_2023_278_MOESM5_ESM.docx]

**Supplementary table 5**. Protein–protein interaction network

| node1 | node2 |
| --- | --- |
| ATP6V0E2 | ATP6V1C1 |
| C9orf72 | LRRK2 |
| DRAM1 | WIPI1 |
| GBA | LRRK2 |
| GNAI3 | PIK3CB |
| GNAI3 | LRRK2 |
| LRRK2 | RALB |
| LRRK2 | PIK3CB |
| SH3GLB1 | WIPI1 |
| WDFY3 | WIPI1 |
