# Supplementary material for: Identification and validation of autophagy-related genes in Kawasaki disease
Source: Hereditas. 2023 Apr 21;160:17. doi: 10.1186/s41065-023-00278-9 (PMC10120123; doi:10.1186/s41065-023-00278-9)
Supplement: Supplementary file 6 — Additional file 6: Supplementary Table 6. mRNA–miRNA interaction network. [file 41065_2023_278_MOESM6_ESM.docx]

**Supplementary table 6.**. mRNA-miRNA interaction network

| node1 | node2 |
| --- | --- |
| ATP6V1C1 | hsa-miR-519b-3p |
| ATP6V1C1 | hsa-miR-421 |
| ATP6V1C1 | hsa-miR-769-5p |
| ATP6V1C1 | hsa-miR-362-3p |
| ATP6V1C1 | hsa-miR-654-3p |
| ATP6V1C1 | hsa-miR-934 |
| ATP6V1C1 | hsa-miR-761 |
| ATP6V1C1 | hsa-miR-3619-5p |
| ATP6V1C1 | hsa-miR-4458 |
| ATP6V1C1 | hsa-miR-4500 |
| C9orf72 | hsa-miR-142-3p |
| C9orf72 | hsa-miR-146b-5p |
| C9orf72 | hsa-miR-513a-5p |
| C9orf72 | hsa-miR-3163 |
| C9orf72 | hsa-miR-4428 |
| CAMKK2 | hsa-miR-133b |
| CAMKK2 | hsa-miR-671-5p |
| CAMKK2 | hsa-miR-760 |
| DRAM1 | hsa-miR-139-5p |
| DRAM1 | hsa-miR-181a-5p |
| DRAM1 | hsa-miR-181b-5p |
| DRAM1 | hsa-miR-181c-5p |
| DRAM1 | hsa-miR-181d-5p |
| DRAM1 | hsa-miR-3612 |
| DRAM1 | hsa-miR-3150b-3p |
| DRAM1 | hsa-miR-4784 |
| EPAS1 | hsa-miR-4306 |
| GNAI3 | hsa-miR-222-3p |
| GNAI3 | hsa-miR-524-5p |
| GNAI3 | hsa-miR-520h |
| GNAI3 | hsa-miR-654-3p |
| GNAI3 | hsa-miR-1276 |
| KLHL3 | hsa-miR-222-3p |
| KLHL3 | hsa-miR-524-5p |
| KLHL3 | hsa-miR-520h |
| KLHL3 | hsa-miR-654-3p |
| KLHL3 | hsa-miR-1276 |
| PLEKHF1 | hsa-miR-4436a |
| QSOX1 | hsa-miR-124-3p |
| RALB | hsa-let-7b-5p |
| RALB | hsa-miR-429 |
| RALB | hsa-miR-1193 |
| RALB | hsa-miR-3179 |
| RALB | hsa-miR-3194-5p |
| RALB | hsa-miR-3064-5p |
| SH3GLB1 | hsa-miR-17-5p |
| SH3GLB1 | hsa-miR-20a-5p |
| SH3GLB1 | hsa-miR-29a-3p |
| SH3GLB1 | hsa-miR-93-5p |
| SH3GLB1 | hsa-miR-106a-5p |
| SH3GLB1 | hsa-miR-129-5p |
| SH3GLB1 | hsa-miR-106b-5p |
| SH3GLB1 | hsa-miR-302a-3p |
| SH3GLB1 | hsa-miR-302b-3p |
| SH3GLB1 | hsa-miR-302c-3p |
| SH3GLB1 | hsa-miR-302d-3p |
| SH3GLB1 | hsa-miR-373-3p |
| SH3GLB1 | hsa-miR-20b-5p |
| SH3GLB1 | hsa-miR-520a-3p |
| SH3GLB1 | hsa-miR-520c-3p |
| SH3GLB1 | hsa-miR-519d-3p |
| SH3GLB1 | hsa-miR-520d-3p |
| SH3GLB1 | hsa-miR-543 |
| WDFY3 | hsa-miR-19a-3p |
| WDFY3 | hsa-miR-330-3p |
| WDFY3 | hsa-miR-519c-5p |
| WDFY3 | hsa-miR-519b-5p |
| WDFY3 | hsa-miR-520c-5p |
| WDFY3 | hsa-miR-518d-5p |
| WDFY3 | hsa-miR-4676-3p |
| WDFY3 | hsa-miR-4726-5p |
