# Supplementary material for: Identification and validation of autophagy-related genes in Kawasaki disease
Source: Hereditas. 2023 Apr 21;160:17. doi: 10.1186/s41065-023-00278-9 (PMC10120123; doi:10.1186/s41065-023-00278-9)
Supplement: Supplementary file 7 — Additional file 7: Supplementary Table 7. mRNA–TF interaction network. [file 41065_2023_278_MOESM7_ESM.docx]

**Supplementary table 7**. mRNA-TF interaction network

| node | node2 | node | node2 |
| --- | --- | --- | --- |
| ATP6V0E2 | FLI1 | GNAI3 | STAT3 |
| ATP6V0E2 | GATA2 | PIK3CB | GATA2 |
| ATP6V0E2 | SNAI2 | PIK3CB | TAL1 |
| ATP6V0E2 | MYC | PIK3CB | TFAP4 |
| ATP6V1C1 | FLI1 | PIK3CB | STAT3 |
| ATP6V1C1 | STAT1 | PIK3CB | TFAP2C |
| ATP6V1C1 | TFAP4 | PIK3CB | TP53 |
| ATP6V1C1 | GABPA | PLEKHF1 | TFAP4 |
| C9orf72 | CREB1 | PLEKHF1 | JUN |
| C9orf72 | E2F4 | PLEKHF1 | FOXM1 |
| LRRK2 | ERG | PLEKHF1 | MITF |
| LRRK2 | MYB | QSOX1 | FLI1 |
| LRRK2 | JUN | QSOX1 | GABPA |
| LRRK2 | GATA2 | QSOX1 | MYC |
| LRRK2 | KLF9 | QSOX1 | RUNX1 |
| LRRK2 | TFAP4 | QSOX1 | TFAP4 |
| LRRK2 | RELA | QSOX1 | GATA1 |
| DEPP1 | TP53 | QSOX1 | AR |
| DEPP1 | FLI1 | QSOX1 | ARNT |
| DEPP1 | FOXM1 | QSOX1 | FOXM1 |
| DEPP1 | ELK3 | QSOX1 | GATA3 |
| DEPP1 | MYC | QSOX1 | JUN |
| DEPP1 | GATA1 | QSOX1 | STAT3 |
| DEPP1 | SP1 | QSOX1 | CDX2 |
| CAMKK2 | TFAP4 | QSOX1 | GATA6 |
| CAMKK2 | GABPA | RALB | TFAP4 |
| DRAM1 | FLI1 | RALB | ELK1 |
| DRAM1 | STAT1 | RALB | JUN |
| DRAM1 | FOXM1 | RALB | SOX2 |
| DRAM1 | GATA2 | RALB | AR |
| DRAM1 | XBP1 | SH3GLB1 | FLI1 |
| DRAM1 | TAL1 | SH3GLB1 | STAT1 |
| DRAM1 | GRHL2 | SH3GLB1 | TFAP2C |
| DRAM1 | BRD4 | SH3GLB1 | ERG |
| DRAM1 | ARNT | TSPO | FLI1 |
| DRAM1 | KLF4 | TSPO | ERG |
| DRAM1 | GATA3 | TSPO | TFAP2C |
| EPAS1 | ESR1 | TSPO | GABPA |
| EPAS1 | FLI1 | TSPO | TFAP4 |
| EPAS1 | TFAP2C | WDFY3 | SOX2 |
| EPAS1 | FOXA1 | WDFY3 | IKZF1 |
| EPAS1 | HSF1 | WDFY3 | GATA3 |
| EPAS1 | GATA2 | WDFY3 | GABPA |
| EPAS1 | CREB1 | WDFY3 | GATA2 |
| EPAS1 | FOXP1 | WDFY3 | FOXP1 |
| EPAS1 | ERG | WDFY3 | TFAP4 |
| EPAS1 | TAL1 | WDFY3 | SRF |
| EPAS1 | ARNT | WDFY3 | STAT1 |
| EPAS1 | MYC | WDFY3 | UBTF |
| EPAS1 | TEAD4 | WDFY3 | MITF |
| EPAS1 | NRF1 | WDFY3 | STAT5A |
| EPAS1 | SP1 | WIPI1 | FLI1 |
| EPAS1 | RNF2 | WIPI1 | RUNX1 |
| EPAS1 | MITF | WIPI1 | MYB |
| FBXL2 | MITF | WIPI1 | TFAP2C |
| FBXL2 | FOXP1 | WIPI1 | NR2F2 |
| FBXL2 | MYC | WIPI1 | FOXA1 |
| FBXL2 | REST | WIPI1 | GABPA |
| GBA | RUNX1 | WIPI1 | FOXM1 |
| GBA | GABPA | WIPI1 | JUN |
| GBA | SP2 | WIPI1 | ATF2 |
| GBA | RAD21 | WIPI1 | KLF9 |
| GNAI3 | ELK1 | WIPI1 | NFYA |
| GNAI3 | STAT1 | WIPI1 | TFAP4 |
| WIPI1 | MYC |  |  |
