# Supplementary material for: Identification and validation of autophagy-related genes in Kawasaki disease
Source: Hereditas. 2023 Apr 21;160:17. doi: 10.1186/s41065-023-00278-9 (PMC10120123; doi:10.1186/s41065-023-00278-9)
Supplement: Supplementary file 8 — Additional file 8: Supplementary Table 8. mRNA–drug network. [file 41065_2023_278_MOESM8_ESM.docx]

**Supplementary table 8**. mRNA-drug network

| gene | drug |
| --- | --- |
| DRAM1 | CISPLATIN |
| LRRK2 | VANDETANIB |
| LRRK2 | PALBOCICLIB |
| PIK3CB | COPANLISIB |
| PIK3CB | MIDOSTAURIN |
| PIK3CB | OCTREOTIDE |
| PIK3CB | IDELALISIB |
| PIK3CB | DOXORUBICIN |
| PIK3CB | LOVASTATIN |
| PIK3CB | ALPELISIB |
